# English Fluency Is Not Enough: My Journey to Cultural Competency

**DOI:** 10.1007/s13187-022-02212-5

**Published:** 2022-08-12

**Authors:** Jinho Jung

**Affiliations:** 1 University of California, San Diego, La Jolla, CA 92903 USA; 2 NAFLD Research Center, Division of Gastroenterology and Hepatology, Department of Medicine, University of California, ACTRI Building, 9452 Medical Center Drive 1W502A11, La Jolla, CA 92037 USA; 3 School of Medicine, University of California at Irvine, Irvine, CA 92617 USA

**Keywords:** API, Asian, Barriers, Cancer, Cultural competency, Disparities, Pacific Islander, Public health

## Abstract

This reflection article shares insight from an immigrant who initially struggled with cultural differences between South Korea and the USA. Through his personal experiences and anecdotes from cross-cultural training, the author describes the importance of cultural competency to effectively communicate and treat diverse patient populations in the USA. Encouraging cultural discussions to recognize obvious and subtle cultural differences may help providers become better advocates for patients.

Have you ever had a stranger ignore your polite “Good Morning!” or rush through the door without a word of thanks as you held it open or push his way onto the elevator? A few years ago, that rude stranger might have been me, feeling equally awkward when receiving and extending such courtesies. Surely, I appeared rude in both circumstances.

Growing up in South Korea until the age of 18, I had learned a very different set of norms. Upon arriving in the USA, I gradually made friends and soon realized that the cultural differences between Korea and the USA were much bigger than I had anticipated. Merely speaking English fluently was not enough to fit into the American culture. I felt the most comfortable eating with chopsticks. When I slurped pasta, frowned eyebrows signaled that I was behaving inappropriately. I was clueless about how to navigate a formal dinner setting.

Navigating the seemingly complex American culture was exhausting. To cope, I searched for a safe place to be truly myself. A university-sanctioned student club, the Asian Pacific Islander (API) Cancer Outreach Team, welcomed me with open arms [1, 2]. Its mission was to reduce cancer morbidity and mortality rates in the API community. The club members were part of a community-campus partnership with Asian grocery stores. The students learned how to share cancer control information with shoppers at the Asian grocery stores, an education strategy with proven efficacy [3].

The club’s mission aligned with my medical school aspirations, while also promising to offer a periodic escape from my cross-cultural exhaustion. Surrounded by Asian Americans from other Asian cultures, I would not have to worry constantly about my unintentional cultural gaffes.

I quickly became dismayed as I realized that the cultural differences among the many API cultures were as great as the cultural differences I was experiencing between Korean and American cultures. Interestingly, the full enlightenment occurred during one of our club’s quarterly potluck meetings. While we all expertly wielded our chopsticks, nabbing even the smallest peas remaining on our plates, it was in our conversations where we shared our differences.

The only rule for the potluck was that we had to bring a dish that we eat at home and a story to share about our culinary offering. Among the most favorite were my bulgogi, Eric brought taro cookies, and Hang many variations of Chinese dumplings, which she taught us to make and had everyone prepare and then cook. The list of potluck options we enjoyed was endless.

Through our simple stories about the food we shared, we also shared information about the rich cultural heritage and personal stories behind them. The floor was open for members to discuss their unique dining rules. For example, in China, the guests do not clean their plates as the hosts might worry that they did not prepare enough food. In Vietnam, after picking up your food from the family-style serving dish, you are expected to put it in your bowl first rather than directly putting it into your mouth. The good dinner combined with robust discussions of cultural humility and multiple humorous anecdotes related to cultural misunderstandings helped the team members to embrace the subtle differences among our API cultures.

Yet, dining etiquettes are only one aspect of cultural differences within different cultures. On a day-to-day basis, I also experienced how Korean and American students behave differently in classroom settings. For example, most Koreans would never ask our professor a question during class for multiple reasons. First, such behavior would be seen as inappropriately trying to stand out amongst peers. Second, Koreans assume asking a clarification question is disrespectful as it is the student’s responsibility to comprehend the materials. Third, Korean students fear that they might be asking a stupid question. As such, exam grades in the Korean education system are often weighed heavily compared to class participation. On the contrary, participation in classroom discussion is encouraged in the USA and one’s culture-based hesitancy to speak can compromise one’s grades. Suddenly, I had to embody behaviors that were uncouth in Korea but were essential to academic success in the USA.

Apparently, this hesitancy to speak up was as universal as our expedient use of chopsticks among fellow Asian American club members. With a few tips and tricks from students who experienced similar challenges, I tried to find a way to break through the dilemma without compromising my cultural values. I would use office hours to ask questions rather than during class to minimize internalized peer pressure to ask “good” questions. Admittedly, the first time I set foot in my biology professor’s office was intimidating, but I quickly realized that he appreciated my questions. For classes that were graded through class participation, I would jot down what I wanted to say to make sure I did not sound or look foolish.

While finding ways to work around the American education system, I began to wonder if the shoppers I was talking with about cancer screening/prevention were equally hesitant to ask me questions. They nodded so politely and with that, I was just assuming their receptivity as evidence of complete comprehension. What if that were not true? Incomplete understanding could be a serious barrier to taking the recommended action on the potentially life-saving information I was sharing with them, like getting a mammogram, getting screened/treated for Hepatitis C or vaccinated for Hepatitis B, and getting screened for colorectal cancer.

Surely, they were not fully comprehending the complex health information without asking a single question. I knew what prevented me from speaking up, so how could we better convey the critical information in ways that worked around that cultural hesitancy issue?

A ruthless review of our education program was encouraging. Our education sessions with Asian grocery shoppers were nearly always accomplished with students from their own culture. All our disseminated brochures and displays were translated into the communities’ diverse Asian languages. We called upon our elder relatives to check the language clarity and accessibility of our translated materials. As a result, our linguistically accessible brochures from students sharing their own culture helped create a safe, friendly environment for shoppers to ask questions and share opinions, behaviors we hoped would invite them to become more proactive in taking care of their health. Once the questions started, the conversations became rich with the essential details they wanted to know. Maybe that’s why our club’s cancer education outreaches to Asian grocery shoppers have been so well received.

Along the way, we also observed differences we could not explain. For example, at Korean grocery stores, shoppers were hesitant to accept brochures to share with their loved ones, while shoppers at the Filipino grocery stores were happy to take brochures to share and even stopped to ask for additional brochures to share during subsequence shopping trips. I wondered whether the predominance of Confucianism within my Korean culture, which emphasizes modesty, might explain the difference with the Filipino community, where the emphasis is on smooth interpersonal relationships. Sometimes, the unexplainable remained just that—observations without explanations. Thus, I have become more cautious about converting what I have experienced into generalizations of effective cancer education strategies for Asian communities. Such generalizations defeat the purpose of cultural sensitivity.

My fellow club members all recognized such cultural differences. We constantly engaged in discussions to find understanding and from there, more effective ways to disseminate cancer-related information across the various API communities. During our weekly meetings, we would stimulate a typical or challenging outreach interaction that had occurred during the previous week to discuss these cultural differences. By recognizing obvious and subtle cultural differences through these simulations, club members focused on helping each other become better healthcare advocates for all API communities. As we interacted with Asian grocery shoppers from cultures different from our own, we saw the impact of tailoring our message to the preference and needs of the communities we were serving. Intimate stories shared by the shoppers helped us stay motivated to continue honing our cross-cultural competency.

The excitement and camaraderie from the club’s shared mission to reduce the burden of cancer within the API community helped me to become fully engaged in the club’s activities. Somewhere in that process of embracing its mission, I suddenly realized that I was no longer feeling alone and out of place. I had also become more comfortable when I encountered people from different cultures. I was becoming a citizen of the world, not just of Korea. The skills I was learning were helping me to learn how to transcend my perceived cultural barriers. I was engaging in civic-minded activities while learning skills of great personal and community benefit, most notably that cultural humility is an essential ingredient of cultural competency.

As such, cultural discussions can teach us important tips and tricks about other cultures. My favorite earliest cultural tip remains, “Double-dipping is not allowed when you are eating chips and dips!” Finally, in closing, when we put an end to cancer, the club members plan to write a cookbook!

## References

[1] Tat J, Nguyen LT, Hung SM, et al (2017) Strategy for sustaining cancer education services for underserved communities. MedSurg Nursing Jan 26:33–38, 43. 30351572

[2] Sadler GR, Nguyen F, Doan Q (1998). Strategies for reaching Asian Americans with health information. Am J Prev Med.

[3] Wang JL, Acevedo N, Sadler GR (2018) using comics to promote colorectal cancer screening in the Asian American and Pacific Islander Communities. Journal of Cancer Education Dec 33(6): 1263–1269 10.1007/s13187-017-1241-4 28646456

